# Tobacco use and household expenditures on food, education, and healthcare in low- and middle-income countries: a multilevel analysis

**DOI:** 10.1186/s12889-015-2423-9

**Published:** 2015-10-31

**Authors:** Young Kyung Do, Mary Ann Bautista

**Affiliations:** Department of Health Policy and Management, Seoul National University College of Medicine and Institute of Health Policy and Management, Seoul National University Medical Research Center, 103 Daehak-ro, Jongno-gu, Seoul, 110-799 Korea; Saw Swee Hock School of Public Health, National University of Singapore, 21 Lower Rd, Singapore, 119077 Singapore

**Keywords:** Tobacco use, Household expenditure, Economic impact, Health and development

## Abstract

**Background:**

The majority of one billion smokers worldwide live in low- and middle-income countries (LMICs) and the highest proportion of smokers in most of these countries belong to the lower socioeconomic groups. This study aimed to investigate the associations between tobacco use within households and expenditures on food, education, and healthcare in LMICs.

**Methods:**

Using data from the World Health Survey, this cross-sectional study included a sample of 53,625 adult males aged <60 years from 40 LMICs. Multilevel, mixed-effects linear regression was used to determine the association between current tobacco use status of the main income provider (daily; occasional; no use) and three categories of (logged) household expenditures: food, education, and healthcare; controlling for age, level of education, household wealth quintile, marital status, urban–rural setting, country-level income group, and region.

**Results:**

In the preferred random-slope models that controlled for covariates, daily tobacco use was associated with lower household expenditures on education and healthcare by 8.0 % (95 % confidence interval: −12.8 to –3.2 %) and 5.5 % (−10.7 to –0.3 %), respectively. The association between tobacco use and food expenditure was inconsistent across models.

**Conclusions:**

Tobacco use in LMICs may have a negative influence on investment in human capital development. Addressing the tobacco use problem in LMICs could benefit not only the health and economic well-being of smokers and their immediate families but also long-run economic development at a societal level.

## Background

Tobacco use is a major public health problem; it is associated with local and systemic health effects [1] and has been documented to contribute to the global non-communicable disease (NCD) epidemic [2]. In fact, 50 % of tobacco users die from tobacco-related causes [3]. As early as the 1990s, tobacco-related mortality had been projected to reach nearly two million annual deaths worldwide [4]. Estimates in 2009 showed that the global smoking prevalences among adult (≥15 years) males and females were 36 and 8 %, respectively [5]. Today, the tobacco epidemic continues to cause over five million deaths each year [3]. If the tobacco problem remains inadequately addressed, annual tobacco-attributable mortality could rise to as high as 9.7 million by 2030 [6]. This mortality effect is not limited to the direct effects of tobacco use on smokers, as tobacco use has negative health effects to non-smokers as well. In fact, secondhand smoke (SHS) exposure is associated with negative health outcomes [1, 7–10]; 10 % of annual deaths worldwide are premature mortality due to exposure to SHS [11].

The high disease burden associated with tobacco use also comes with a large economic toll on individual users and the society at large [12–14], especially in low- and middle-income countries (LMICs) [5, 15, 16]. A number of studies have presented estimates of the overall economic impact of tobacco use on a specific country [17–21] or specific population groups within a country or state [22, 23]. There are varied mechanisms through which smoking generates a negative economic impact, one of which involves the direct medical costs of tobacco-related morbidity, disability, hospitalizations, and medical consultation fees [14, 18, 19, 21, 24–26]. Depending on the level of pooled healthcare financing available, the direct medical costs of smoking represent an important financial externality: the direct healthcare costs that smokers inflict on society are partly paid for by the entire population, including non-smokers [27]. Another mechanism that could explain the negative economic impact of smoking refers to the indirect costs due to lost productivity from smoking-related illnesses and disability at the individual level, which can also reduce tax revenues and ultimately impact national economic growth [14, 22, 23, 28]. Tobacco consumption has become more than just a health concern and is, in fact, a development issue [29]. The Bellagio statement on tobacco and sustainable development explicitly highlighted that “tobacco is a major threat to sustainable and equitable development” [30], and the tobacco-poverty link has increasingly been recognized in the global tobacco control community [31, 32]. The profound impact of smoking on the health and economic well-being of society warrants its integration into the global development agenda [29, 31, 32], especially because population health and economic development are inextricably intertwined [33].

While the economic costs of tobacco use are typically estimated based on national-level parameters of medical costs and productivity loss attributable to tobacco-related diseases, there is another important but less visible micro-level mechanism through which tobacco use can have negative consequences for the economic well-being of smokers and their immediate family members. This negative impact has been described as the “crowding-out effect” of tobacco use on household consumption [12, 13, 34–37]. Given a fixed household budget, spending on tobacco use may divert household economic resources from essential items, such as food, education, and healthcare, which are indispensable components of human development, particularly for young children. Theoretically, the magnitude and the mechanisms of the crowding-out effect of tobacco use on food, education, and healthcare may vary. This effect is likely to be smaller on food compared with education and healthcare given the less discretionary nature of food consumption. Unlike its impact on household expenditures on food and education, however, the association between tobacco use and healthcare expenditures is ambiguous. In theory and as reported in previous studies, tobacco use is positively associated with healthcare spending; healthcare expenditures can be higher among households with tobacco users due to the direct health-related costs of tobacco-related diseases [17, 34, 38]. On the other hand, the income effect of tobacco use on healthcare spending may also work in the opposite direction as tobacco use may diminish disposable income for healthcare [13, 34]. Hence, the direction and magnitude of the resulting association between tobacco use and healthcare expenditure depends on the relative magnitude of these two counteracting effects.

Within the specific context of LMICs, the high smoking prevalence among males in lower-income groups [16, 39] may be crucial in shrinking a family’s resources allocated for other key expenditures to a large extent [12, 13]. This phenomenon is especially relevant because despite observing a growing number of dual-income households in many societies worldwide, males remain as the major household income provider in LMICs [39]. Moreover, the majority of the world’s one billion smokers live in LMICs [3] and the highest proportion of smokers in most of these countries belong to the lower socioeconomic groups [27]. These observations have critical implications considering that individuals from LMICs are expected to forego essential spending than their richer counterparts [12]. In fact, lower-income households have been reported to use a significant and greater portion of their income on smoking [40] – a portion of an already limited income which could have otherwise been allotted for the important necessities. Although a previous study in India has shown a lack of a statistically significant difference in the association of smoking on expenditures for other goods across regions and income groups [34], research findings in Taiwan, China, and Bangladesh [12, 13, 20] suggest that lower-income households remain at risk for the crowding-out effect of household smoking on spending for other basic needs. These concerns are fundamental to the endeavour of describing the long-run economic impact of smoking in LMICs.

As in most studies on the economic impact of tobacco use, the association of smoking with reduced spending for other goods, such as food, education, healthcare and other necessities, has been investigated in studies using data from several individual countries: India [34], China [13, 37], Cambodia [36], and Taiwan [12]. Along these lines, the current study exploits multi-country data from the World Health Survey (WHS) collected in a cross-nationally comparable format, to examine the association between household tobacco consumption among male income providers and three categories of household expenditures, thereby improving the generalizability of the key study findings. Furthermore, the study uses a statistical approach that accounts for country-level heterogeneity in a multilevel regression framework. Specifically, the hypothesis that current tobacco use of the main income provider within households is negatively associated with household expenditures on food, education, and healthcare is tested. As described in the foregoing theoretical prediction of this association, the magnitude of the said negative association is greater for education expenditure compared with food; the association is ambiguous for healthcare expenditure.

## Methods

### Data sources and study participants

The WHS, an initiative of the World Health Organization (WHO), was conducted in 2002–2004 to gather a comprehensive set of baseline data on the health of populations [41], including information on the current functional and monitoring capacity of health systems, and the outcomes related to investments in health systems [41, 42]. In the WHS, each WHO region included a varying number of participating countries, ranging from 18 (AFRO) to 4 (EMRO). The WHS questionnaire consists of five modules covering the (1) population health status; (2) risk factors (e.g., tobacco and alcohol); (3) health system responsiveness; (4) coverage; and (5) healthcare expenditures, which can be conducted separately. Each participating country can choose from these modules and decide on the most practical and cost-effective survey method (i.e., household face-to-face survey, computer assisted telephone interview, or computer assisted personal interview) [41]. The three-part WHS is administered in each country’s respective vernacular and consists of a household questionnaire, an individual questionnaire, and a section on vignettes; information on the household expenditures and smoking status are drawn from the first two sections respectively. As of this writing, data collection and preliminary tabulation of WHS results have been completed in 70 countries; data are publicly available for analysis [43]. Primarily aimed to provide valid, reliable and comparable data at a minimal cost, the WHS results are expected to provide policymakers with the necessary evidence to guide their decision-making strategies and programmes [42]. Additional country-level data on the purchasing power parity (PPP) conversion factor for private consumption per USD (i.e., household final consumption expenditure) are obtained from the World Bank database of economic indicators [44]. The target population of the WHS consists of all adults (i.e., at least 18 years old, male and female) living in private households, including household members who are in an institution due to a health condition. Individuals and households from other non-household living arrangements such as military reservations or group lodgings are excluded [45, 46].

The current cross-sectional study focused on a sample of adult male respondents aged <60 years from 43 LMICs in the WHS (*N* = 93,744). Observations from Ecuador (*n* = 2,540) and two other countries (i.e., Bosnia, Zimbabwe) (*n* = 1,775) without data on wealth indicators and PPP respectively were excluded. Those with any missingness for the explanatory variables and covariates were also excluded (*n* = 15,773) (i.e., complete case analysis). Focusing on the male respondents who are the main household income providers, 22,571 observations were further excluded. The final working sample consisted of 53,625 individuals from 40 LMICs in the WHS.

### Variables

#### Household expenditures

The key outcome variables included three categories of household expenditures: food, education, and healthcare. The corresponding variables for the household expenditures were derived from item responses related to the amount of household spending on: ‘food, including such things as [rice], meat, fruits, vegetables, and cooking oils…include the value of any food that was produced and consumed by the household, and exclude alcohol, tobacco and restaurant meals; education fees and supplies; and healthcare costs, excluding any insurance reimbursements,’ respectively reported in the household expenditure section of the WHS household questionnaire. The expenditure values, reported in the local currencies, were divided by the consumption-specific PPP exchange rate [44] to obtain the common currency equivalent valuation of goods that can be consumed within each country. The natural logarithm of the expenditure values were then used so that the incremental change in the explanatory variable can be interpreted as a percentage change in the expenditure values.

#### Tobacco use

The main explanatory variables included dummy variables for the three categories of current tobacco use status (i.e., ‘Daily’, ‘Yes, but not daily’, and ‘No, not all’ as the omitted reference category) determined based on individual respondents’ responses to the survey question: ‘Do you currently smoke any tobacco products such as cigarettes, cigars, or pipes?’.

#### Covariates

Potential confounding factors that may influence the relationship between the outcomes and the main explanatory variables are included as covariates (i.e., the respondent’s age, level of education, household wealth quintiles, marital status, urban–rural setting, country-level income group, and the corresponding WHO region groupings). Based on country-level income, the 40 LMICs included in the analysis were divided into two income groups: poorer (21) and less poor (19). The wealth indicators were derived from responses to questions about the home, referring to previous works that used principal components analysis in constructing income indices [47, 48].

### Statistical analysis

Linear regression was used to determine the association between the main explanatory variables of current tobacco use status and each of the three outcomes of household expenditures; estimating both unadjusted and adjusted models for each. The adjusted model controlled for other factors that may influence the association between tobacco use and household expenditures. To accommodate the multilevel nature of the data (i.e., individual observations are nested within countries), multilevel analysis was conducted.

Three different estimation methods were used depending on the key assumptions on country-level influences. The fixed-effects model was estimated to control for country-level unmeasured differences that might affect the association between smoking and the expenditure variables; the random-intercept model was also estimated considering that such country-level unmeasured differences may be random in nature. The Hausman test was then used to determine the preferred estimation method between the fixed-effects and random-intercept models. The fixed-effects model would be favored if the null hypothesis of the test was rejected (i.e., not controlling for the country-level unmeasured differences would lead to inconsistent estimates in the random-intercept model, because such country-level differences may confound the association between tobacco use and household expenditures). Alternatively, if the null hypothesis was not rejected, the random-intercept model would be preferred on the basis of efficiency.

Once the test has established that the random-intercept model should be favored over the fixed-effects model, a further consideration was determining whether the influence of tobacco use can be better estimated by incorporating a random component that accounts for country-level heterogeneity (i.e., a random-slope model where the influence of tobacco use may vary across countries), rather than a fixed estimate of tobacco use alone as assumed in the random-intercept model. A likelihood-ratio (LR) test would then guide the selection of the preferred estimation strategy. Rejecting the null hypothesis of the LR test would suggest that the random-slope model was favored over the random-intercept model that only accounted for the shifts in country-level intercepts. In both the unadjusted and adjusted models, the total number of observations in each of the three household expenditure categories varied due to missing observations in the relevant dependent variables.

Finally, the country-specific point estimates of daily tobacco use and their 95 % confidence intervals were derived from the preferred models and presented in graphs. All statistical analyses were conducted in Stata version 12.0, with the main procedures xtmixed and xtreg used for multilevel regression analysis [49].

## Results

Summary statistics of the study sample show the distribution of respondents and crude prevalences of tobacco use across the different categories of explanatory variables (Table 1). Approximately 31.1 % of respondents received no formal education or less than primary level education. In terms of current tobacco use, roughly 31.8 % and 11.3 % of the sample of male adults reported daily and occasional tobacco use respectively. A relatively higher prevalence of tobacco use was reported among respondents with a lower level of education compared with those who completed at least high school (41.27 %). The tobacco use prevalence was found to be highest in the poorest quintile at 49.3 % (vs. 36.0 % in the wealthiest quintile). In the same vein, the crude tobacco use rate in less poor LMICs was shown to be relatively higher than that in the poorer LMICs (44.0 % vs. 42.1 %).

**Table 1:** Summary statistics for the study sample

Variables	Unweighted mean or % distribution	Crude tobacco use prevalence^a^ (%)
Tobacco use		—
No use	56.87	
Daily	31.82	
Occasional	11.30	
Age (in years), mean	38.7	—
Level of education		
High school/College/University/Postgraduate degree completed	22.08	41.27
Secondary completed	25.57	41.43
Primary completed	21.23	45.37
No formal education or lower than primary level	31.13	44.30
Household wealth quintile		
5^th^ (highest)	19.10	35.95
4^th^	19.64	39.98
3^rd^	19.84	44.12
2^nd^	20.14	45.52
1^st^ (lowest)	21.28	49.27
Marital status		
Currently married	77.71	44.07
Never married	10.19	37.53
Separated/Divorced/Widowed	4.80	48.25
Co-habiting	7.29	37.52
Urban–rural setting		
Urban/Semi-urban	47.04	41.02
Rural	52.96	45.00
Country-level income group^b^		
Less poor (Lower-middle and upper-middle income)	54.91	43.95
Poorer (Low income)	45.09	42.12
Region		
Africa	28.36	26.40
Americas	29.24	35.97
Eastern Mediterranean	5.25	47.00
Europe	5.59	59.94
Southeast Asia	16.33	59.91
Western Pacific	15.23	62.50

*Notes*: The study sample consists of male respondents (age < 60) who are the main household income providers from low-and middle-income countries in the World Health Survey (WHS), *N* = 53,625. ^a^Prevalence of daily and occasional tobacco use combined. ^b^Country-level income group classification was based on data from The World Bank (Fiscal Year 2004) which corresponds to WHS data for calendar year 2002. High-income countries were excluded

The unadjusted linear regression model demonstrated statistically significant associations of both daily and occasional tobacco use of the main male income provider with decreased household expenditures on food, education, and healthcare in most of the estimation methods used (Table 2), although the preferred estimation method indicated by the Hausman test varied depending on the outcome of interest.

**Table 2:** Unadjusted regression model of logged household expenditures for food, education and healthcare

	Food			Education			Healthcare		
Variables	Random-slope	Random-intercept	Fixed-effects	Random-slope	Random-intercept	Fixed-effects	Random-slope	Random-intercept	Fixed-effects
	(1)	(2)	(3)	(4)	(5)	(6)	(7)	(8)	(9)
Current tobacco use (*ref. No use*)									
Daily	−0.064**	−0.051**	−0.052**	−0.208**	−0.183**	−0.184**	−0.137**	−0.121**	−0.122**
Occasional	−0.083**	−0.050**	−0.050**	−0.138**	−0.065**	−0.066**	−0.093*	−0.043	−0.043
Constant	4.666**	4.657**	4.656**	1.517**	1.498**	1.547**	1.584**	1.574**	1.455**
*SD (Daily)*	.081 (.015)^a^	—	—	.137 (.027)^a^	—	—	.148 (.028)^a^	—	—
*SD (Occasional)*	.103 (.029)^a^	—	—	.120 (.035)^a^	—	—	.143 (.044)^a^	—	—
*SD (Constant)*	.517 (.058)^a^	.520 (.059)^a^	—	.699 (.079)^a^	.691 (.078)^a^	—	.662 (.075)^a^	.652 (.074)^a^	—
Hausman test^b^	—	[*χ* ^c^(2) = 4.05 (p = 0.132)]		—	[*χ* ^c^(2) = 7.59 (p = 0.023)]		—	[*χ* ^c^(2) = 5.82 (p = 0.055)]	
Likelihood ratio test^c^	[*χ* ^c^(2) = 59.86 (p < 0.001)]		—	[*χ* ^c^(2) = 43.86 (p < 0.001)]		—	[*χ* ^c^(2) = 47.16 (p < 0.001)]		—
Observations (countries)	53,185 (40)			50,732 (40)			50,602 (40)		

*Notes:* **p < 0.01, *p < 0.05; SD, standard deviation of estimated coefficient; ^a^standard error in parentheses; ^b^Rejecting the null hypothesis would favor fixed-effects to random-intercept; ^c^Rejecting the null hypothesis would favor random-slope over random-intercept

In the adjusted regression model (Table 3), daily tobacco use of the main male income provider was associated with an increase in household expenditure on food by 2.4 − 2.8 % (Table 3, Columns 1–3). In contrast, the association between daily tobacco use and household expenditure on education and healthcare was negative and statistically significant in all estimation methods, albeit with a considerably smaller magnitude than the unadjusted model. Based on the LR test and the Hausman tests results, our preferred estimation was random-slope estimation for education and healthcare expenditures. The statistical tests provided less conclusive guidance for the preferred estimation method for food expenditures than for the other two household expenditures, which is also reflected by the small difference in magnitude of the coefficients on daily tobacco use across the three estimation methods (0.024, 0.028, and 0.028). Results of the preferred random-slope estimation showed that, daily tobacco use was statistically significantly associated with an 8.0 % and 5.5 % reduction in household spending on education and healthcare, respectively (Table 3, Columns 4 and 7), the magnitude of which was larger than that from the random-intercept model (Table 3, Columns 5 and 8) and the fixed-effects model (Table 3, Columns 6 and 9). In the preferred random-slope models of education and healthcare expenditures, substantial country-level variations were observed for the coefficient estimates for tobacco use status. Occasional tobacco use was not statistically significant in any of the models estimated.

**Table 3:** Adjusted regression model of logged household expenditures for food, education and healthcare

	Food			Education			Healthcare		
Variables	Random-slope	Random-intercept	Fixed-effects	Random-slope	Random-intercept	Fixed-effects	Random-slope	Random-intercept	Fixed-effects
	(1)	(2)	(3)	(4)	(5)	(6)	(7)	(8)	(9)
Current tobacco use (*ref. No use*)									
Daily	0.024*	0.028**	0.028**	−0.080**	−0.068**	−0.067**	−0.055*	−0.045*	−0.045*
Occasional	−0.020	−0.011	−0.011	−0.014	0.006	0.006	−0.028	−0.002	−0.002
Age (in years)	0.029**	0.029**	0.029**	0.164**	0.164**	0.164**	0.001	0.001	0.001
Age^c^/100	−0.032**	−0.032**	−0.031**	−0.184**	−0.184**	−0.184**	0.003	0.003	0.003
Level of education *(ref. At least high school)*									
Secondary completed	−0.076**	−0.076**	−0.076**	−0.100**	−0.100**	−0.098**	−0.067**	−0.067**	−0.066**
Primary completed	−0.110**	−0.111**	−0.111**	−0.258**	−0.258**	−0.257**	−0.154**	−0.156**	−0.155**
No formal education/lower than primary	−0.158**	−0.158**	−0.158**	−0.449**	−0.450**	−0.448**	−0.283**	−0.284**	−0.284**
Household wealth quintile *(ref. 5* ^*th*^ *, highest)*									
4th	−0.269**	−0.269**	−0.269**	−0.542**	−0.542**	−0.542**	−0.250**	−0.250**	−0.250**
3rd	−0.384**	−0.385**	−0.385**	−0.762**	−0.762**	−0.763**	−0.352**	−0.352**	−0.353**
2nd	−0.506**	−0.506**	−0.507**	−0.943**	−0.944**	−0.945**	−0.448**	−0.449**	−0.450**
1st (lowest)	−0.710**	−0.711**	−0.712**	−1.150**	−1.152**	−1.154**	−0.621**	−0.626**	−0.627**
Marital status *(ref. Currently married)*									
Never married	−0.279**	−0.278**	−0.279**	−0.451**	−0.451**	−0.451**	−0.360**	−0.361**	−0.361**
Separated/Divorced/Widowed	−0.347**	−0.347**	−0.347**	−0.554**	−0.555**	−0.554**	−0.324**	−0.327**	−0.326**
Co-habiting	−0.058**	−0.058**	−0.058**	−0.169**	−0.170**	−0.170**	−0.059	−0.060*	−0.061*
Rural (*vs Urban/Semi-urban*)	−0.206**	−0.207**	−0.207**	−0.107**	−0.108**	−0.109**	0.002	0.002	0.002
Poorer LMICs (*vs Less poor LMICs*)	−0.085	−0.084	—	−0.676**	−0.678**	—	0.057	0.082	—
Constant	4.472**	4.470**	4.631**	−0.542**	−0.534**	−0.858**	1.685**	1.677**	1.880**
*SD (Daily)*	.035 (.016)^a^	—	—	.095 (.025)^a^	—	—	.113 (.026)^a^	—	—
*SD (Occasional)*	.049 (.036)^a^	—	—	.056 (.041)^a^	—	—	.101 (.049) ^a^	—	—
*SD (Constant)*	.416 (.047)^a^	.416 (.047)^a^	—	.426 (.049)^a^	.431 (.049)^a^	—	.534 (.061) ^a^	.531 (.060)	—
Hausman test^b^	—	[*χ* ^c^(15) = 24.11 (p = 0.063)]		—	[*χ* ^c^(15) = 16.22 (p = 0.368)]		—	[*χ* ^c^(15) = 19.94 (p = 0.174)]	
Likelihood ratio test^c^	[*χ* ^c^(2) = 3.46 (p = 0.177)]		—	[*χ* ^c^(2) = 9.01 (p = 0.011)]		—	[*χ* ^c^(2) = 17.71 (p < 0.001)]		—
Observations (countries)	53,185 (40)			50,732 (40)			50,602 (40)		

*Notes:* **p < 0.01, *p < 0.05; SD, standard deviation of estimated coefficient; ^a^standard error in parentheses; ^b^Rejecting the null hypothesis would favor fixed-effects to random-intercept; ^c^Rejecting the null hypothesis would favor random-slope over random-intercept. Country-level income group classification was modified based on data from The World Bank (Fiscal Year 2004) which corresponds to WHS data for calendar year 2002. Region dummies were included but are not presented here

Both education and household wealth exhibited strong associations with all three categories of household expenditures. Compared with the reference category of respondents who have completed at least high school, individuals with lower levels of education showed a larger magnitude of decrease in consumption on food, education, and healthcare. Household wealth also showed positive associations with all three categories of expenditures, in a consistent gradient across quintiles. Although living in a rural area was consistently associated with a statistically significant decrease in expenditures on food and education, a larger magnitude of this reduction was observed in expenditure on food (20.6 %). Notably, compared with the less poor LMICs, living in a poorer LMIC was associated with a decrease in spending on education by 67.6 %; its associations with household expenditures on food and healthcare were not statistically significant.

**Fig. 1:**
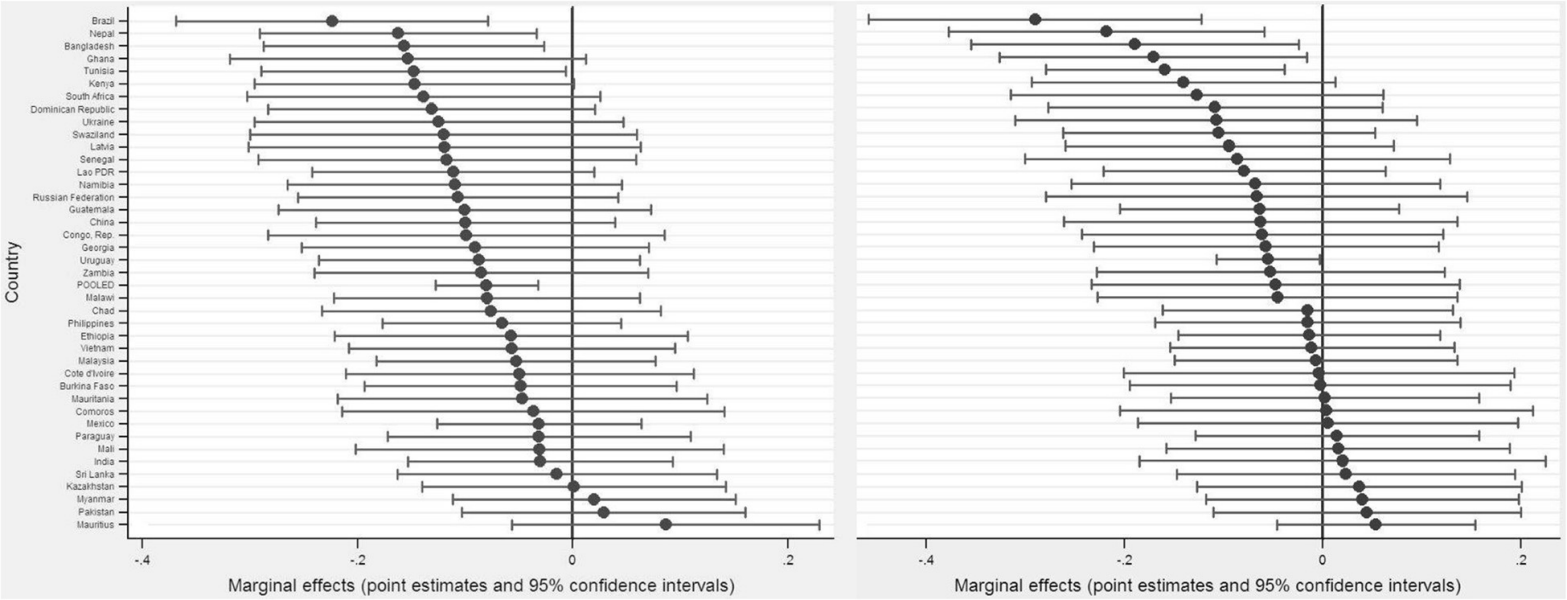
Daily tobacco use is associated with reduced household expenditures on education and healthcare. Left: Household expenditure on education, pooled estimate: decreased by 8.0 % (CI: −12.8 to –3.2 %). Right: Household expenditure on healthcare, pooled estimate: decreased by 5.5 % (CI: −10.7 to –0.3 %); the point estimates of the random-slope model of logged household expenditures on education and healthcare at 95 % CI are illustrated as black dots, and estimates whose CI cross the vertical line (*zero*) are not statistically significant

Despite considerable cross-country variations, the negative associations between daily tobacco use and household expenditures on education and healthcare were consistent across the majority of LMICs in the study (Fig. 1).

## Discussion

In LMICs, daily tobacco use of the main male income provider was negatively associated with household expenditures on education and healthcare, even after adjusting for socioeconomic characteristics. The magnitude of the associations in the adjusted models was smaller than in unadjusted models but was still not trivial (i.e., 8.0 % and 5.5 % reduction in expenditures on education and healthcare, respectively). The association between tobacco use and food expenditure was inconsistent, depending on whether or not socioeconomic characteristics were controlled for. Unlike daily tobacco use, occasional tobacco use was statistically insignificant in all adjusted models. Accounting for country-level heterogeneity in the random-slope models did not change the key results qualitatively but increased the magnitude of the estimated associations of daily tobacco use with household expenditures on education and healthcare. In line with the theoretical framework and the policy implications of this study, several key findings merit further discussion.

Consistent with findings from previous country-level studies [13, 34], a negative association of tobacco use with household expenditure on education was also reported in the current study. Importantly, the decreased expenditure on children’s education in households whose main income providers are daily tobacco users draws attention to the possible intergenerational effect described in previous studies [13, 34], where tobacco use exacts its potentially critical long-run impact on human capital investment and economic development. This negative association is critical given that decreased spending on education has been associated with increased poverty [50] and that improving the quality and quantity of education had a wider impact on the economy [51]. This particular study finding may also be explained by the higher time preference of smokers [52]; being less future-oriented, smokers may place a lower value on the future benefits of education, which potentially influences their decision to reduce spending on education.

Compared with household expenditures on education, understanding the negative association between tobacco use and healthcare spending may be less straightforward. Although previous research on the crowding-out effect of tobacco use has reported a negative association between tobacco use and household expenditure on healthcare [13, 34], the current study findings differ from other studies that reported higher direct and indirect healthcare costs in households with tobacco users [53, 54]. Given the likely increase in the demand for healthcare due to tobacco-related illnesses in households with tobacco users, the negative association between tobacco use and healthcare spending suggests that the potential crowding-out effect of tobacco use dominates in the current study. Healthcare, unlike food, is subject to consumption that is more discretionary in nature; it is possible that households with smokers choose to forego spending on treatment for illnesses for reasons that cannot be identified in the study. This finding has implications for an individual’s future earning potential, given the evidence of the influence of childhood health on a person’s earning and career pathways as an adult [55, 56]. Distinguishing between the direct medical costs due to tobacco-related diseases and healthcare expenditures in general, may tease out healthcare spending for specific tobacco-related diseases and possibly allow for a more meaningful interpretation of the study findings. Nevertheless, the observed negative association of daily tobacco use with healthcare may be due to the potentially limited supply of healthcare services in LMICs, which may preclude a greater likelihood of spending on healthcare.

Although previous studies reported that tobacco use crowds out household spending on food particularly in LMICs [13, 34, 36], the current study found a negative association only in the unadjusted model for daily tobacco use. Once socioeconomic characteristics were controlled for in the adjusted model, a positive association was found between daily tobacco use and household expenditures on food. This rather unexpected finding deviates from our theoretical prediction that tobacco use is negatively associated with household expenditure on food. Several possible arguments may help explain the positive association between daily tobacco use and food expenditures in the adjusted model. First, food – a less discretionary good – remains a basic need that must be satisfied regardless of the additional expenses borne from tobacco use, suggesting that the crowding-out effect of tobacco use on food, if any, may be less than the observed effect on other discretionary goods. Second, as in the argument for the association between tobacco use and household expenditures on education, the higher time preference associated with tobacco use [52] may also be relevant in explaining the observed association. Relative to education and healthcare, nourishment is considered as an immediate need and therefore remains a priority for smokers who highly value their present and immediate utility. Finally, assuming that both tobacco and food are obtained from the same commercial source such as the marketplace, households with smokers may then have an increased exposure to food products and may consequently spend more on food.

These study findings are germane to the persistent and growing tobacco epidemic in LMICs. Overall, the results of this study contribute to better understanding the micro-level mechanisms at work in the long-run consequences of tobacco use on economic development in LMICs. Specifically, the negative association between daily tobacco use and household expenditures on education and healthcare draws important implications on human capital investment [50, 51, 55, 56]. These households with smokers are shown to invest less in education, which presents a missed opportunity to improve future employment prospects for the children in these households had they received better education. The reduced spending on healthcare also suggests that family members in households with a daily smoker in LMICs may experience even greater unmet health needs.

The percentage of daily tobacco users in the study sample of male adults from LMICs (31.8 %) remains high. Furthermore, previous studies have demonstrated that poverty does not inhibit poor individuals from consuming tobacco and other smokeless tobacco products [57] and that the poor are still more likely to consume tobacco than the rich [27, 58]. Indeed, smoking continues to play an important role in the cycle of poverty and poor health; not only does it contribute to persistent impoverishment, but it considerably widens the socioeconomic inequalities in LMICs as well [17, 38]. In summary, this study highlights the evolution of tobacco smoking from being a major global health concern to being an equally important development issue. Considering tobacco use as a health and development issue may then provide additional support for advocating policy interventions to address the global tobacco epidemic.

The key findings of the study must be considered with important caveats. Foremost, causal inference is precluded by the cross-sectional study design and its inability to control for possible unobserved heterogeneity between households with tobacco users and those without. Furthermore, the operational definition of household tobacco use was made using only the information available on the main male income provider of the household. The amount of household spending on tobacco and the composition of spending for other components of the household budget could not be determined because the male main income provider was the only smoker identified within households. Nevertheless, markedly different results between daily and occasional tobacco use – a proxy for the household burden of tobacco use – suggests a dose–response relationship, where greater spending on tobacco use (i.e., daily tobacco use) may lead to a greater reduction in household expenditures on education and health. In addition, limiting the study population to the sample of adult males who are the main income providers may not capture the effect of the smoking prevalence and labor force participation rates among women, which have been shown to increase in recent years [39, 59]; excluding this group in the sample could have biased the effect of smoking on household expenditures. Aside from using self-reported measures, which may be subject to social desirability and recall bias, using the individual smoking status of the main income provider and the pooled household income as the key independent and dependent variables respectively may also limit the interpretation of the results. Future research may then consider other factors that are beyond the scope of the currently available data (e.g., household composition, the number of children in the household) to develop a better understanding of the relationship between individual smoking status and household resource allocation patterns.

These limitations should nonetheless be viewed in the context of the main strength of the study. The study used a comprehensive dataset from multiple LMICs with a standardised questionnaire and thus improved the generalisability of the study findings. To this end, the study used estimation methods that accounted for the multilevel nature of the data.

## Conclusion

Tobacco use in LMICs is negatively associated with household expenditures on education and healthcare, suggesting the potential negative influence of tobacco use on investment in human capital development. Addressing the tobacco use problem in LMICs could benefit not only the health and economic well-being of smokers and their immediate families but also long-run economic development at a societal level.
